# Case Report: Rapid resolution of psychogenic cough in an adolescent following bromazepam treatment

**DOI:** 10.3389/fpsyt.2026.1920653

**Published:** 2026-09-24

**Authors:** Mansour Alharbi

**Affiliations:** Department of Psychiatry, College of Medicine, Qassim University, Burayidah, Saudi Arabia

**Keywords:** adolescent psychiatry, benzodiazepines, bromazepam, case report, habit cough, psychogenic cough, somatic cough syndrome, somatoform disorders

## Abstract

**Background:**

Somatic cough syndrome, formerly termed psychogenic cough or habit cough, is an under-recognized cause of chronic cough in pediatric populations, characterized by repetitive, dry, and honking cough that is absent during sleep. Behavioral interventions are first-line treatment with high success rates of 80%–90%. However, when access to behavioral therapy is unavailable, alternative pharmacological interventions, particularly benzodiazepines, have received minimal investigation in this population.

**Case presentation:**

I report a 13-year-old previously healthy male patient with abrupt-onset, near-continuous dry cough occurring every 2 seconds during waking hours. The cough had a distinctive honking quality and disappeared completely during sleep. The patient presented with severe impairment in function, including inability to eat for 3 days, multiple emergency presentations requiring intravenous hydration, and complete school absence. Medical evaluation, including physical examination, laboratory investigations, spirometry, and chest X-ray, was unremarkable. Empirical trials of inhaled bronchodilators and nasal corticosteroids yielded no improvement. Psychiatric assessment excluded psychotic features, depression, obsessive-compulsive disorder, and tic disorder but noted family stressors, including high expressed emotion and overprotective parenting behavior. Based on the sign of sleep-related cough cessation and exclusion of organic pathology, somatic cough syndrome was diagnosed. Given the severity of impairment and family circumstances precluding immediate behavioral therapy, bromazepam 3 mg orally was initiated. Within 24–36 hours, the cough resolved completely. At 3-month follow-up, the patient remained symptom-free without medication. He had returned to school and demonstrated normal eating patterns.

**Conclusion:**

This case demonstrates rapid and sustained resolution at 3-month follow-up of severe psychogenic cough following short-term bromazepam treatment in an adolescent. While behavioral therapy is still an evidence-based first-line treatment, this report suggests using benzodiazepines as a pharmacological intervention alternative when behavioral therapy is unavailable. The rapid response and sustained remission at 3-month follow-up are consistent with the hypothesis that benzodiazepines may interrupt the self-perpetuating cycle of anxiety and cough in somatic cough syndrome. Further research is needed for this case in pediatric populations to establish efficacy, safety, and optimal treatment duration.

## Introduction

Cough is the most frequent presenting complaint in pediatric clinical practice. Most cough episodes are self-limited and caused by respiratory infections. A proportion of children develop chronic cough that defies conventional medical explanation. The clinicians must consider a functional cause, including somatic cough syndrome, when clinical evaluation and investigation reveal no identifiable organic cause of cough. This condition, which was previously called psychogenic cough or habit cough, represents a significant diagnostic and therapeutic challenge in pediatric and adolescent medicine (1).

Somatic cough syndrome is a chronic cough arising in the context of somatic symptom disorder, without identifiable structural or physiological pathology. The American College of Chest Physicians (ACCP) adopted this term in 2015 to replace psychogenic cough, aligning practice with the Diagnostic and Statistical Manual of Mental Disorders, Fifth Edition (DSM-5), which had removed the word psychogenic from its nosology (2). Despite this change, clinical recognition remains limited because many children endure prolonged delays in diagnosis, unnecessary investigations, and repeated failed treatments before consideration of functional etiology (3).

The condition mainly affects patients under 18 years of age (4). In the cohort of Wright and Balfour-Lynn (5), nine in ten children had already received a median of three pharmacological trials before the diagnosis was established. A two-decade series of 140 children by Weinberger et al. (6) showed a male predominance (58%), a median age of 10 years, and a mean symptom duration of 4 months; the associated functional burden, including school absence and repeated emergency visits, was substantial.

Among all clinical features, the nocturnal disappearance of the cough stands out as the most diagnostically informative: the consistent absence of cough during sleep reliably separates functional from structural causes and is considered pathognomonic. This nocturnal pattern was corroborated in the series by Wright and Balfour-Lynn (5), where sleep-related cough cessation was documented in 93% of affected children. The cough presents as a dry, high-frequency, honking sound during waking hours that tends to worsen when the child feels that others are paying attention to it (7) (8).

Treatment has traditionally focused on behavioral interventions: suggestion therapy (9), cognitive behavioral therapy, and psychoeducation. Unfortunately, access to trained therapists remains limited in many settings, creating a therapeutic gap in which pharmacological alternatives have received limited investigation. Bromazepam, a medium-potency benzodiazepine (10, 11), exerts its effects via gamma-aminobutyric acid type A (GABA-A) receptor modulation, producing anxiolytic and sedative properties relevant to functionally mediated somatic disorders. In a controlled series of 301 patients presenting with somatized psychogenic disorders, Podiwinsky and Jellinger (10) found that bromazepam at 3 mg administered three times daily produced a favorable response (rated excellent or good) in nearly all cases (94%), with an acceptable tolerability profile. Calandrino et al. (12) documented a complete resolution of anxiety-driven somatic symptoms in a 10-year-old within 48 hours of initiation of bromazepam, suggesting utility in functionally mediated pediatric presentations.

To the best of our knowledge, no published report has specifically documented the use of bromazepam for somatic cough syndrome in an adolescent. This case addresses that gap, illustrating the potential of short-course benzodiazepine therapy as a pharmacological bridge when behavioral therapy is unavailable.

## Case description

### Presenting complaint and history

A 13-year-old male adolescent, previously healthy and with normal development, was brought by his father to the pediatric clinic complaining of a 1-week history of a dry cough that was abrupt in onset and continuous. The cough had a distinctive staccato, honking quality, and occurred approximately every 2 seconds during waking hours. The symptom led to marked distress and significant functional impairment. The patient had not eaten for the previous 3 days because of persistent cough and had made multiple visits to the emergency department for intravenous fluid administration. He had been absent from school for the entire duration of the illness. The complete clinical timeline, including all investigations, interventions, and follow-up contacts, is summarized in **Table 1**.

**Table 1:** Clinical timeline of the case.

Timepoint	Clinical event	Key finding/status	Intervention
Day 0 (Onset)	Abrupt onset of honking cough (~every 2 s)	Near-continuous cough; absent during sleep; no fever or wheeze	None
Days 1–5 (Pre-referral)	Multiple emergency department visits; school absence; unable to eat	Intravenous hydration required; bronchodilators and nasal corticosteroids failed	Short-acting beta-2 agonist inhaler and nasal corticosteroid; no response
Day 7 (Pediatric clinic)	Referral for organic workup	Forced expiratory volume in 1 second/forced vital capacity ratio 92%; chest X-ray normal; complete blood count, erythrocyte sedimentation rate, C-reactive protein, and immunoglobulin E normal	Provisional diagnosis: somatic cough syndrome; referral to psychiatry
Day 7 (Psychiatric assessment)	Psychiatric evaluation	No psychosis, depression, obsessive-compulsive disorder, or tic features; high expressed emotion in the family noted	Diagnostic formulation; psychoeducation; reassurance
Day 7 (Treatment initiation)	Bromazepam 3 mg once daily started (off-label)	Off-label consent obtained from guardian	Bromazepam 3 mg orally once daily
Day 9 (Day 2 of treatment)	Telephone follow-up	Cough completely ceased within 24–36 h; eating resumed; returned to school	Continue bromazepam
Day 16 (1 week post-cough resolution)	Telephone follow-up	Cough absent; full function restored	Begin 5-day taper and discontinuation
Day 21 (Taper complete)	Medication discontinued	No rebound; no withdrawal symptoms	Bromazepam discontinued
Month 3 (Follow-up)	Telephone follow-up	Fully cough-free; school examinations completed; no somatic recurrence	None; behavioral therapy referral declined by family

The father emphasized that the cough disappeared completely during sleep and had started suddenly without preceding prodromal symptoms. There were no associated symptoms, including rhinorrhea, nasal congestion, sore throat, fever, wheezing, dyspnea, or chest pain. He had no history of atopy, rhinitis, or bronchial asthma, no recent exposure to environmental irritants or passive smoke, and no recent travel.

When asked about potential psychosocial precipitants, the father denied any clear stressors at home or school. He noted that the patient had school examinations during this period but characterized these as routine. The father described the examinations as “nothing more than usual.” There was no recent family discord, bullying, peer conflict, or academic failure, and the patient’s academic performance was above average. No previous history of psychiatric illness, behavioral difficulties, or functional somatic symptoms was identified.

### Medical evaluation

A thorough systemic examination was conducted by a pediatrician; inspection of the ear, nose, and throat was unremarkable. There was no tonsillar enlargement, postnasal drip, or pharyngeal erythema, and the nasal mucosa appeared normal. The lungs were clear bilaterally on respiratory examination, without wheeze, crackles, or reduced air entry. The cardiovascular examination was normal, with a regular rhythm, no murmurs, and normal peripheral perfusion. Abdominal examination and neurological assessment were unremarkable.

Laboratory investigations were conducted to exclude infectious, inflammatory, and allergic etiologies. Complete blood count (CBC) results and serum immunoglobulin E levels were within normal limits, and there was no elevation of the erythrocyte sedimentation rate (ESR) or C-reactive protein. Spirometry demonstrated a forced expiratory volume in 1 second (FEV1)/forced vital capacity (FVC) ratio of 92% predicted, which was within normal limits, and a posteroanterior chest X-ray was interpreted by a pediatric radiologist as demonstrating no parenchymal abnormality, pleural effusion, or mediastinal pathology.

Empirical pharmacological treatment had been initiated over the preceding 5 days. Bronchodilator inhalers (short-acting beta-2 agonists) and nasal corticosteroids had been administered without any clinical improvement. The negative organic workup, absence of treatment response, and complete sleep-related cough cessation supported a provisional diagnosis of psychogenic cough (somatic cough syndrome). The pediatrician referred the patient to the psychiatrist for more assessment and management.

### Psychiatric assessment

The psychiatric evaluation was conducted in an outpatient setting. The patient was fully conscious, oriented, and cooperative, but the cough, occurring every 2 seconds throughout the interview, severely limited direct history taking. The cough was dry, staccato, and honking, with no premonitory urge or tic-like feature. The majority of the clinical history was obtained from the father.

The father presented with high expressed emotion and overprotective behavior, denying any identifiable psychosocial stressors despite acknowledging concurrent school examinations.

On mental status examination, no psychosis, major depressive episode, obsessive-compulsive features, or anxiety disorder was observed. Family psychiatric history was negative for tic spectrum disorders or somatoform disorders.

The diagnostic formulation favored somatic cough syndrome over transient tic disorder given the absence of a premonitory urge, lack of tic waxing and waning, a uniformly high repetition rate (every 2 seconds), and a negative family history of tic spectrum disorders.

### Treatment and outcome

Following the diagnostic formulation, psychoeducation was provided to both the patient and his father. The concept of functional cough was explained in accessible language, with reassurance that the condition carried an excellent prognosis and did not indicate serious pathology.

No appropriately trained behavioral therapist was available at this time. Given severe functional impairment (3 days without eating, multiple emergency department visits, and complete absence from school), pharmacological intervention was initiated.

Based on the published efficacy data for bromazepam in somatized psychogenic disorders (10), bromazepam was initiated at a once-daily dose of 3 mg. The off-label nature was discussed with the father, and written informed consent was obtained. Potential adverse effects and a planned short-course taper were outlined.

At 2-day telephone follow-up, the father reported complete cough cessation within 24–36 hours, full resumption of eating, and return to school; no adverse effects were observed. At 1-week review by phone, the cough remained absent; bromazepam was tapered and discontinued over 5 days without rebound. At 3-month follow-up, the patient remained entirely cough-free, having completed school examinations successfully.

## Discussion

### Diagnostic considerations and nomenclature

The nomenclature for functional cough has evolved significantly. The renaming was deliberate: as Vertigan (13) explains, the ACCP chose “somatic cough syndrome” partly to reduce the stigma attached to the word “psychogenic,” while simultaneously bringing the diagnosis into alignment with DSM-5 terminology. Even so, Wang et al. (14) caution that neither term is underpinned by a universally agreed definition or a validated diagnostic criterion set, and a degree of conceptual ambiguity persists in the literature. In this report, both terms are used interchangeably to reflect the transitional state of the literature.

As Vertigan (13) notes, psychological comorbidity is not exclusive to somatic cough and may co-occur across a range of cough etiologies, where it modulates symptom experience and renders rigid diagnostic categorization difficult. In the present case, the acute onset, short duration, and absence of identifiable psychiatric comorbidity are best characterized as an acute somatic cough episode in an otherwise psychiatrically healthy adolescent.

### Pathognomonic features and differential diagnosis

The complete absence of cough during sleep was the key diagnostic anchor in this case, a pathognomonic sign that reliably separates functional from structural cough. Sleep cessation was confirmed in 93% of cases (51 of 55) by Wright and Balfour-Lynn (5), who regarded nocturnal inquiry as the single most decisive question in clinical evaluation. Ambulatory cough monitoring in a 13-year-old independently corroborated this day–night discrepancy, with near-zero cough frequency during sleep hours (7). Liu et al. (15) describe the phenotype as a dry, honking, repetitive cough that diminishes with distraction and resolves in sleep, features fully exhibited in the present case.

Organic causes requiring exclusion include asthma, allergic rhinitis, gastroesophageal reflux, infections, and foreign body aspiration, all excluded in this case by spirometry, chest radiography, and laboratory investigations.

Among functional diagnoses, somatic cough syndrome must be distinguished from tic cough. Key distinguishing features of tic cough, including a premonitory sensory urge, the capacity for voluntary suppression, and a fluctuating course, were highlighted by Karakaya and Şişmanlar (16) as criteria that differentiate tic cough from somatic cough. Elangovan (17) reported a case of chronic cough absent during sleep that was ultimately diagnosed as vocal tics and treated with risperidone. In the present case, a uniformly high frequency, absence of a premonitory urge, no other tics, and a negative family history favored a somatic diagnosis. The rapid response to an anxiolytic rather than an antipsychotic is consistent with a somatic cough diagnosis; however, treatment response alone should not be considered confirmatory evidence, and this interpretation requires cautious qualification as anxiolytic agents may also produce symptomatic relief in tic-related cough presentations.

### Established treatment approaches

The evidence base is dominated by behavioral interventions. Suggestion therapy produced striking results in the study by Lokshin et al. (9): every child treated experienced cessation during the session itself, and more than four in five remained cough-free at extended follow-up. These outcomes were independently replicated by Weinberger et al. (6) across a larger cohort of 140 patients, with 95% achieving cough cessation within a single 15–30-minute therapeutic encounter. Other documented approaches include cognitive behavioral therapy (18), behavior modification (4), and self-hypnosis (19).

Using reassurance alone, Wright and Balfour-Lynn (5) achieved full symptom resolution in 82% of their cohort; notably, parental acceptance of the functional diagnosis was a powerful predictor of outcome, with resolution rates of 96% among accepting parents compared with only 54% among those who remained skeptical.

Despite this relatively favorable evidence landscape for behavioral interventions, a significant practical barrier exists. Appropriately trained therapists are not uniformly available across all clinical settings. This resource gap is particularly pronounced in developing countries and rural or semi-urban areas. The present case illustrates this reality. The patient was experiencing severe functional impairment, including inability to eat, multiple emergency presentations, and school absence, yet no behavioral therapist was accessible within a clinically acceptable timeframe.

### Rationale for bromazepam in somatic cough syndrome

Benzodiazepines attenuate cough through modulation of the nucleus tractus solitarius (NTS), the principal brainstem integration center for afferent cough reflex arcs. GABA-A receptor agonism at the NTS suppresses sensitized central cough pathways by a mechanism that is distinct from peripheral airway bronchodilation, and is therefore particularly relevant when cough persists in the absence of identifiable organic pathology. In somatic cough syndrome, in which cortical amplification and conditioned reflex circuits sustain cough after resolution of any peripheral trigger, GABA-A-mediated central inhibition provides a mechanistically coherent pharmacological rationale for benzodiazepine therapy.

Within the benzodiazepine class, bromazepam was selected on the basis of three converging considerations. First, the only controlled series documenting benzodiazepine efficacy in somatized presentations specifically evaluated bromazepam at 3 mg three times daily across 301 patients (10), providing the most robust published evidence base for this agent in functionally mediated somatic disorders. Second, Draper (11) described a beneficial clinical response to bromazepam in a functionally mediated somatic syndrome, further supporting its utility in this indication. Third, Calandrino et al. (12) documented complete resolution of anxiety-driven somatic symptoms in a 10-year-old child within 48 hours of bromazepam initiation, providing direct pediatric precedent for rapid functional remission. Bromazepam was also the benzodiazepine available in the local formulary at the time of the patient’s presentation, making it the pragmatically appropriate choice. Together, published controlled evidence, direct pediatric precedent, and local formulary availability jointly justified its selection over alternative members of the class.

The pharmacological rationale rests on converging evidence, and the most extensive evidence base for bromazepam in somatized presentations comes from Podiwinsky and Jellinger (10), whose evaluation across 301 patients yielded favorable outcomes in 94% of cases; symptoms of a psychogenic cardiovascular, gastrointestinal, or anxious nature responded most consistently. Tolerability was good: side effects were limited to mild fatigue or transient vertigo in roughly one in ten patients, and no signs of dependence emerged even when treatment extended beyond 34 months.

Of particular relevance, Podiwinsky and Jellinger (10) noted that bromazepam’s anxiolytic action appeared to facilitate sleep onset as a secondary effect, an observation that may be mechanistically pertinent to somatic cough syndrome, given that the symptom is uniquely absent during sleep. Modulation of arousal states may plausibly interrupt the wakefulness-dependent cycle sustaining the cough.

Calandrino et al. (12) reported a pediatric case of a 10-year-old girl with anxiety-driven hyperventilation syndrome whose symptoms resolved within 48 hours. It is important to note, however, that the treatment protocol in that case included concurrent intravenous midazolam, intravenous electrolyte supplementation, and fluid hydration in addition to oral bromazepam; the therapeutic response therefore cannot be attributed to bromazepam alone. This case provides indirect, hypothesis-generating evidence consistent with a possible anxiolytic effect in functionally mediated pediatric respiratory presentations, but cannot be interpreted as confirmatory evidence for bromazepam efficacy in somatic cough syndrome given the confounded treatment protocol.

Barkin et al. (1) recommended benzodiazepines for psychogenic non-cardiac chest pain, and Draper (11) described bromazepam as “most likely to be effective in the relief of visceral manifestations of anxiety.” Systematic review evidence further supports benzodiazepine efficacy in functional respiratory symptoms (20).

The conservative dose of 3 mg once daily represents approximately one-third of the total daily dose employed by Podiwinsky and Jellinger (10) in adults. This conservative approach was guided by four considerations: (i) the patient’s adolescent age and likely lower body weight relative to adult subjects in the reference study; (ii) the minimum effective dosing principle for pediatric and adolescent pharmacotherapy; (iii) the goal of preserving daytime cognitive function and avoiding excessive sedation during school attendance; and (iv) the brevity of the planned course, which reduced the clinical importance of achieving maximum plasma steady-state concentrations. The dose and its rationale were discussed with the patient’s guardian during the informed consent process. The rapid response within 24–36 hours is consistent with the pharmacokinetic profile of bromazepam (peak plasma concentration 1–4 h, elimination half-life 12–20 h).

### Pediatric safety considerations

Bromazepam is not licensed for pediatric use in most regulatory frameworks, and its prescription in this adolescent was accordingly off-label. Before treatment initiation, the risk-benefit profile including the rationale for a short, self-tapering course was discussed explicitly with the father as legal guardian, and written informed consent for both off-label use and publication was obtained. The dose of 3 mg once daily represents approximately one-third of the adult therapeutic dose reported by Podiwinsky and Jellinger (10), minimizing cumulative benzodiazepine exposure while retaining the anxiolytic effect required to interrupt the conditioned cough reflex arc. Clinical monitoring at each contact encompassed sedation, ataxia, paradoxical disinhibition, and respiratory depression; none of these adverse effects were observed during the 5-day treatment course or at the 3-month follow-up contact.

### Comparison with existing literature

The 24–36-hour timeline to complete cough cessation is notably swift. For context, suggestion-based behavioral approaches have produced cessation within minutes in controlled settings; in-session resolution was reported by Lokshin et al. (9), and Weinberger et al. (6) recorded resolution within a 15–30-minute window. Yet in a naturalistic reassurance-based cohort, Wright and Balfour-Lynn (5) observed that fewer than one in eight patients recovered on the day of consultation, with the majority taking days to weeks to become symptom-free. In contrast, patients without targeted intervention experienced approximately 6 months of continued symptoms (21).

The lack of adverse effects is consistent with published safety data (10). The successful taper without rebound supports short-course utility. Across a mixed pharmacological and psychotherapeutic cohort, Bhatia et al. (3) recorded remission or improvement in 87.5% of patients. The full remission achieved in the present case may partly reflect its acuity, with a 1-week illness duration in contrast to the chronic presentations (median diagnostic delay of 4 years) described by Bashtawi et al. (2). Veeraraghavan et al. (22) reported similar rapid resolution following timely diagnosis, supporting the prognostic importance of early intervention.

### Family dynamics and psychosocial context

The psychosocial context is important to consider. The father’s high expressed emotion and overprotective behavior are documented risk factors in pediatric somatic presentations (23). School examinations, though described as routine, may have served as a precipitating stressor; in a series of 32 children, Bhatia et al. (3) found that school avoidance and attention-seeking behavior were the most frequently identified psychosocial triggers. Parental belief in the diagnosis significantly influences outcomes (5), and the father’s willingness to accept the functional formulation likely contributed to the favorable result.

### Limitations

As a single case report, findings cannot be generalized, and the rapid response may reflect individual characteristics, acute presentation, or non-specific therapeutic effects rather than a direct pharmacological action of bromazepam.

Formal psychometric assessment was not performed (e.g., with the Screen for Child Anxiety Related Emotional Disorders [SCARED] questionnaire), limiting characterization of underlying psychological mechanisms. A placebo effect cannot be excluded; given that reassurance-based approaches yield resolution in up to 82% of patients (5), it is not possible in a single uncontrolled case to attribute the recovery specifically to bromazepam rather than to the non-specific therapeutic effects of the clinical encounter.

Follow-up was limited to 3 months by telephone. Longer-term outcomes regarding potential tic disorder development or other functional diagnoses cannot be evaluated (5). Bromazepam use in adolescents is off-label; its safety profile in this age group has not been established through randomized trials.

No validated patient-reported outcome instrument was administered at baseline or at follow-up; all outcome data rested exclusively on caregiver report and clinician observation. Instruments such as the Leicester Cough Questionnaire or the Pediatric Quality of Life Inventory (PedsQL) would have provided standardized, objective quantification of cough burden and health-related quality of life, enabling meaningful comparison across cases and facilitating future meta-analytic synthesis. Their incorporation is strongly recommended for future case reports in this area.

A follow-up period of 3 months conducted by telephone is insufficient to characterize the long-term trajectory of somatic cough syndrome, the durability of benzodiazepine-facilitated remission, or the potential for symptom substitution with alternative somatic presentations. Future reports should incorporate structured in-person or telemedicine follow-up of at least 12 months, with repeat psychometric evaluation and cough frequency monitoring, to establish whether short-course benzodiazepine therapy produces durable remission or merely defers symptom expression.

### Clinical implications and recommendations

Several clinical implications emerge in this case. Inquiry about sleep cessation should anchor history-taking as the primary diagnostic discriminator (15). Where behavioral therapy is unavailable, short-course bromazepam 3 mg once daily with a planned taper offers a practical pharmacological bridge. Psychosocial assessment, including family dynamics, should inform follow-up even when no clear precipitant is identified. Controlled trials comparing bromazepam, suggestion therapy, and placebo are needed, and longitudinal surveillance is warranted given the documented risk of subsequent tic disorders (9).

## Conclusion

This case report documents the rapid and complete recovery of psychogenic cough (somatic cough syndrome) in a 13-year-old male patient following a short course of bromazepam 3 mg orally. The patient presented with severe functional impairment, including inability to eat, multiple emergency presentations requiring intravenous hydration, and school absence. Comprehensive medical evaluation excluded organic pathology. The pathognomonic feature of complete cough cessation during sleep, combined with the honking character and high frequency of the cough, confirmed the functional diagnosis. Psychiatric assessment excluded major psychiatric comorbidity and tic disorder.

Bromazepam was initiated because of the unavailability of local behavioral therapy resources. Complete cough cessation occurred within 24–36 hours, with no observed adverse effects. Successful taper and discontinuation were achieved within 5 days without symptom rebound. At 3-month telephone follow-up, sustained resolution was confirmed; however, this duration is insufficient to establish long-term outcomes or to exclude delayed recurrence, and extended surveillance with structured clinical reassessment is recommended.

This case adds to the limited pharmacological literature on somatic cough syndrome in adolescents, suggesting bromazepam may be a viable option when behavioral interventions are inaccessible, consistent with published data from Podiwinsky and Jellinger (10) and Calandrino et al. (12).

The limitations of this single case report are obvious: the inability to exclude placebo effects, absence of formal psychometric assessment, and lack of controlled comparison preclude definitive conclusions. Controlled studies comparing short-course benzodiazepine therapy with behavioral interventions and placebo are warranted.

The broader clinical message is one of diagnostic confidence and therapeutic pragmatism. Somatic cough syndrome is recognizable and treatable. Clinicians should be empowered to make this diagnosis on clinical grounds, provide clear explanation to families, and initiate timely intervention to minimize the substantial functional burden this condition imposes.

## Patient perspective

Direct communication with the patient was severely limited at the time of clinical evaluation, as the cough occurring every two seconds throughout the interview precluded sustained verbal exchange; the clinical history was accordingly obtained predominantly from the father as legal guardian. The patient appeared visibly distressed and accepted the functional formulation when it was explained in accessible language by the clinician. At three-month follow-up, the father reported that the patient had fully resumed school attendance, had performed successfully in school examinations, and had returned to normal eating and social functioning, with no residual cough. No structured patient-reported outcome measures, such as the Leicester Cough Questionnaire or the Pediatric Quality of Life Inventory, were employed at baseline or follow-up; the absence of such instruments is acknowledged as a limitation, and their incorporation is recommended for future case reports in this area. Written informed consent for publication was obtained from the father as legal guardian.

## Data Availability

The original contributions presented in the study are included in the article/supplementary material. Further inquiries can be directed to the corresponding author.
